# A Micromachined Silicon-on-Glass Accelerometer with an Optimized Comb Finger Gap Arrangement

**DOI:** 10.3390/mi15091173

**Published:** 2024-09-22

**Authors:** Jiacheng Li, Rui Feng, Xiaoyi Wang, Huiliang Cao, Keru Gong, Huikai Xie

**Affiliations:** 1 School of Integrated Circuits and Electronics, Beijing Institute of Technology, Beijing 100081, China; 3120225693@bit.edu.cn (J.L.); xiaoyiwang@bit.edu.cn (X.W.); 3120231352@bit.edu.cn (K.G.); 2 East China Institute of Photo-Electron lC, Bengbu 233030, China; richardfeng85@gmail.com; 3 Chongqing Institute of Microelectronics and Microsystems, Beijing Institute of Technology, Chongqing 400000, China; caohuiliang1986@126.com; 4 Engineering Research Center of Integrated Acousto-Opto-Electronic Microsystems, Ministry of Education of China, Beijing 100081, China

**Keywords:** MEMS, accelerometer, SOG, comb finger design

## Abstract

This paper reports the design, fabrication, and characterization of a MEMS capacitive accelerometer with an asymmetrical comb finger arrangement. By optimizing the ratio of the gaps of a rotor finger to its two adjacent stator fingers, the sensitivity of the accelerometer is maximized for the same comb finger area. With the fingers’ length, width, and depth at 120 μm, 4 μm, and 45 μm, respectively, the optimized finger gap ratio is 2.5. The area of the proof mass is 750 μm × 560 μm, which leads to a theoretical thermomechanical noise of 9 μg/√Hz. The accelerometer has been fabricated using a modified silicon-on-glass (SOG) process, in which a groove is pre-etched into the glass to hold the metal electrode. This SOG process greatly improves the silicon-to-glass bonding yield. The measurement results show that the resonant frequency of the accelerometer is about 2.05 kHz, the noise floor is 28 μg/√Hz, and the nonlinearity is less than 0.5%.

## 1. Introduction

MEMS accelerometers have been widely used in automotive, navigation, vibration monitoring, and even portable electronic devices [1,2,3]. Among various types of accelerometers, capacitive micromechanical accelerometers are the most widely used [4]. Compared with piezoresistive and piezoelectric types, capacitive accelerometers have many unique advantages, such as a better signal-to-noise ratio, high sensitivity, low drift, and low temperature sensitivity [5].

In most application scenarios, there is a requirement for high sensitivity and the miniaturization of accelerometers [6]. The sensing capacitance of the comb finger structure obtained by surface machining is small, which affects the further improvement of the sensitivity and resolution of the comb finger micromechanical sensors. In order to improve the sensitivity and resolution of micromechanical sensors, capacitive accelerometers with unequal finger gap structures are generally fabricated using bulk silicon processing methods.

Among the bulk silicon processing methods, the silicon-on-glass (SOG) process based on anodic wafer bonding is frequently used to make MEMS accelerometers [7,8,9]. However, in the SOG method, a metal layer between the glass and the silicon wafer is needed to provide electrical connection, making the anodic bonding’s yield not high [10]. At the same time, when the bonding area between the silicon and glass is too small, the bond strength will decrease, and the risk of accelerometer failure will increase.

In addition, in the capacitive accelerometer structure with unequal finger gaps, the distance between the rotor finger and the stator finger is very close, generally less than 10 μm, so the two stator fingers on both sides of each rotor finger cannot be bonded to separate electrodes. Thus, all of the stator fingers are bonded on the same electrode. The gaps of a rotor finger to its two adjacent stator fingers are different with the finger gap ratio as 1:10 or 1:5 [11,12]. This design only considers the capacitance formed on the smaller gap while ignoring the capacitance on the larger gap [13]. Increasing the finger gap ratio will increase the capacitance change per finger pair, but this will increase the area occupied by each finger pair. Thus, there exists an optimal finger gap ratio. However, this optimal ratio is yet seen in the literature.

Therefore, in this work, an asymmetric comb-drive capacitive accelerometer with the optimal finger gap ratio is proposed. The gap ratio optimization is based on maximizing the sensing capacitance in a given area, leading to increased sensitivity and/or reduced chip size. At the same time, an improved SOG process with high manufacturing yield is developed. The accelerometer has been successfully fabricated and tested with a phase-locked amplification circuit. The structural design, circuit design, and fabrication process are presented in Section 2. The characterization results of the fabricated accelerometer are described in Section 3.

## 2. Design and Modeling

### 2.1. Structure Design

**Figure 1:**
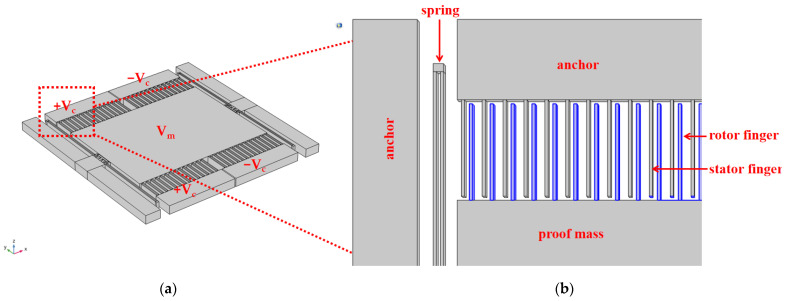
(**a**) Stereo structures of the accelerometer; (**b**) detail of the accelerometer structures.

**Figure 2:**
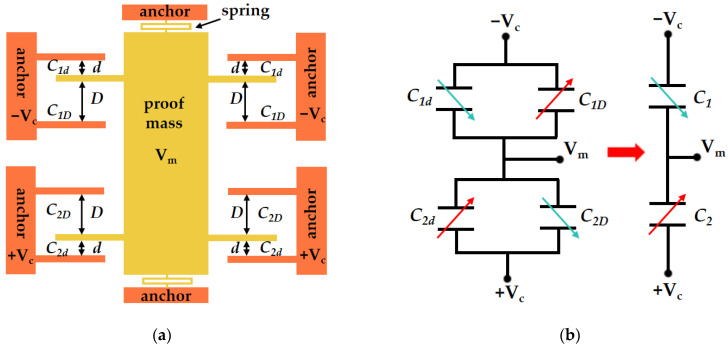
(**a**) The accelerometer structure topology; (**b**) equivalent circuit diagram of the accelerometer.

The 3D model of the accelerometer designed in this paper is illustrated in Figure 1. The proof mass is supported by two springs: the rotor comb fingers (blue) are integrated with the proof mass and move in tandem with it, while the stator comb fingers (gray) are affixed to the silicon anchors. Figure 2a demonstrates that the gaps of a rotor finger to its two adjacent stator fingers are different, where single crystal silicon (SCS) is used as the electrode material. All the fingers are categorized into four distinct groups. The stator fingers symmetrical around the *x*-axis are loaded with the same voltage, and the rotor fingers have the same potential. Consequently, these configurations can ultimately be simplified into two sensing capacitances, as depicted in Figure 2b. The two sensing capacitances, *C*_1_ and *C*_2_, exhibit equal values, and with the movement of the proof mass, one capacitance value increases while the other diminishes, thus generating a differential capacitance that significantly mitigates common mode interference. To optimize the variation of the capacitance values in correlation with displacement, it is essential to design the finger gap *d* and the anti-finger gap *D* with precision.

When the external acceleration is zero, the sensing capacitance of the comb-drive accelerometer can be expressed as follows:

(1) $$C_{1d}=C_{2d}=N\frac{\varepsilon L_{\mathrm{finger}}T}{d}$$

(2) $$C_{1D}{=C}_{2D}=N\frac{\varepsilon L_{\mathrm{finger}}T}{D}$$

where *N* is the number of groups of comb finger, the expression is

(3) $$N=\frac{L_{\mathrm{mass}}}{2W_{\mathrm{finger}}+d+D}$$

The parameter *L_finger_* denotes the length of the finger, *W_finger_* denotes the length of the finger, and *L*_mass_ represents the length of the mass, both of which are constants. The variable *T* signifies the thickness of the accelerometer structure, which can be articulated as

*T* = *r⋅d* (4)

where *r* represents the etching aspect ratio, i.e., the ratio of the vertical etching depth to the lateral etching width in the process of dry etching. Implementing a high aspect ratio allows for maximizing the sensing capacitance density.

When subjected to non-zero external acceleration, the rotor finger experiences a displacement, denoted as *x*. The sensing capacitance of the comb finger accelerometer can be articulated as follows:

(5) $$C_{1d}=N\frac{\varepsilon L_{\mathrm{finger}}T}{d+x}$$

(6) $$C_{2d}=N\frac{\varepsilon L_{\mathrm{finger}}T}{d-x}$$

(7) $$C_{1D}=N\frac{\varepsilon L_{\mathrm{finger}}T}{D-x}$$

(8) $$C_{2D}=N\frac{\varepsilon L_{\mathrm{finger}}T}{D+x}$$

The change in capacitance is expressed as

(9) $$\Delta C=C_{2}-C_{1}=\left({\;C}_{2d}+{\;C}_{2D}\right)-\left({\;C}_{1d}+{\;C}_{1D}\right)$$

The capacitive sensitivity is expressed as

(10) $$S_{c}=\frac{\Delta C}{x}$$

We designate *p* as the ratio of the anti-finger comb gap to the finger comb gap, and the formulation is as follows:

(11) $$p=\frac{D}{d}$$

By substituting formulas (3)–(9) and (11) into (10), we receive

(12) $$S_{c}=r\varepsilon L_{\mathrm{finger}}\left(\frac{L_{\mathrm{mass}}}{2w_{\mathrm{finger}}+d+\mathrm{pd}}\right)\left(\frac{d}{d-x}+\frac{d}{\mathrm{pd}-x}-1-\frac{1}{p}\right)/x$$

**Figure 3:**
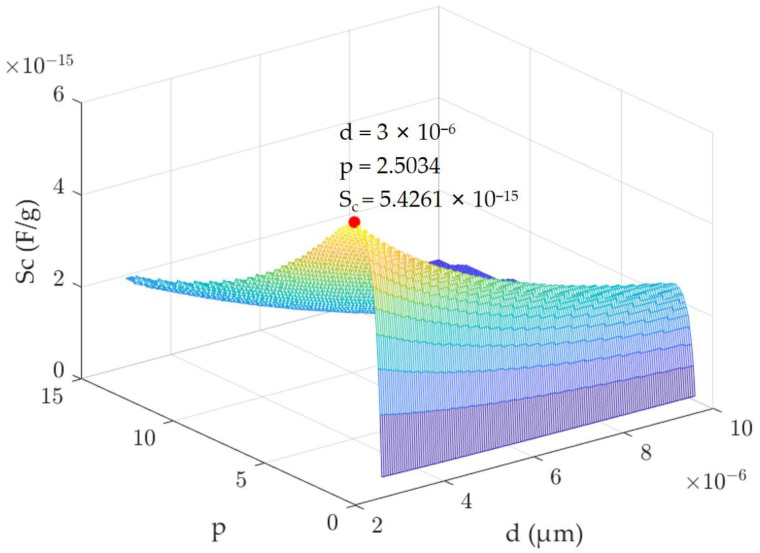
The relationship between the capacitive sensitivity and the parameters *d* and *p* in the case of a certain chip area.

Within the specified chip area, we analyzed the correlation between the capacitive sensitivity and the parameters *d* and *p*, subsequently creating a functional relationship diagram (Figure 3).

Our findings reveal two significant trends. First, with *p* held constant, the capacitive sensitivity increases as the comb finger gap of the accelerometer decreases. Second, with *d* held constant, there is a peak in the capacitive sensitivity at a specific *p* value. Figure 3 highlights the position at which this maximum capacitive sensitivity occurs.

The relationship between capacitance change and acceleration can be obtained by integrating an electromechanical interface within COMSOL and employing a parametric scanning technique. To validate the optimization theory of finger gap that we proposed, we conducted a series of control experiments. In COMSOL, we developed multiple three-dimensional models of comb finger accelerometers, all occupying the same chip area, maintaining a finger gap of 3 µm, consistent structural thickness, and identical spring stiffness, with the primary variable being the ratio of the anti-finger gap *D* to the finger gap *d*.

Ultimately, the capacitive sensitivity for each model was determined through simulation. As detailed in Table 1, when the ratio of the anti-finger gap *D* to the finger gap *d* is 2.5, the capacitive sensitivity reaches its peak. A minor discrepancy exists between the simulation outcomes and theoretical calculations, attributable to the latter’s neglect of fringe capacitance [14].

In summary, as the etching aspect ratio of the etching system employed in this work is about 15, for optimal structural design, a finger gap of 3 μm and a structural thickness of 45 μm are selected [15]. Furthermore, the ratio of the anti-finger gap to the finger gap stands at 2.5, which maximizes capacitive sensitivity within the same chip area.

The torsional spring possesses much smaller stiffness along the sensing axis than those in the other two axes to minimize cross-axis sensitivity. The spring’s stiffness in the sensing axis is readily given by

(13) $$k_{\mathrm{spring}}=\frac{E}{{\left(n-1\right)}^{3}}{\left(\frac{W_{\mathrm{spring}}}{2L_{\mathrm{spring}}}\right)}^{3}T$$

The parameter *E* represents the Young’s modulus of the material, *n* represents the number of turns of the spring, *W_spring_* represents the width of the spring, *L_spring_* represents the length of the spring, and *T* represents the thickness of the spring.

The mechanical sensitivity of the accelerometer is expressed as follows:

(14) $$\frac{x}{a}=\frac{m}{k_{\mathrm{spring}}}$$

The parameter *m* represents the weight of the proof mass.

The mechanical resonant frequency of the accelerometer is then given by

(15) $$f\;=\frac{1}{2\pi }\sqrt{\frac{k_{\mathrm{spring}}}{m}}$$

By considering the available etching aspect ratio, bandwidth, sensing range, and mechanical sensitivity of the accelerometer, the values of the structural parameters of the final accelerometer design are listed in Table 2.

### 2.2. Signal Sensing Circuit Design

In many application contexts, the acceleration being measured typically manifests as a low-amplitude DC signal or a slowly varying signal. To mitigate the detrimental effects of 1/f noise and the DC offset drift of the amplifier—such as temperature-induced drift in the input voltage of operational amplifiers—a modulator or chopper is commonly employed to transform the low-frequency signal into a high-frequency AC signal for amplification and further processing. In practical scenarios, the carrier frequency typically exceeds 20 times the frequency of the modulated signal. Yet, if the carrier frequency becomes excessively high, it imposes greater demands on the bandwidth and slew rate of both operational and instrumentation amplifiers in the circuit. Additionally, the impact of parasitic inductance and parasitic capacitance on the PCB will also increase significantly.

**Figure 4:**
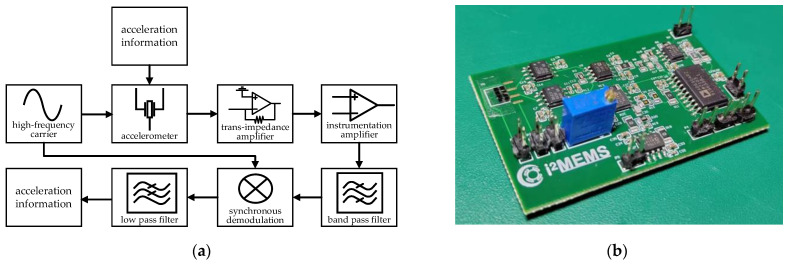
(**a**) Block diagram of the phase-locked amplifier circuit; (**b**) actual manufactured PCB board.

The signal-to-noise ratio is enhanced through the use of a band-pass filter, which effectively suppresses broadband noise, followed by synchronous demodulation and low-pass filtering to obtain an amplified acceleration signal. This methodology is referred to as the phase-locked amplifier circuit [16]. The block diagram of the circuit scheme is shown in Figure 4a. The physical layout of the PCB is depicted in Figure 4b. The trans-impedance amplifier circuit employs an OP657 operational amplifier chip, featuring a gain bandwidth product of 1.6 GHz and an input voltage noise of 4.8 nV/Hz^1/2^. The instrument amplifier is an AD8421 chip, which has a 2 MHz bandwidth and a common mode rejection ratio (CMRR) of 94 dB. The demodulation circuit utilizes an AD630 chip.

### 2.3. Fabrication Process

In this optimized SOG procedure, 6-inch BF33 glass wafers (Schott, Mainz, Germany) are utilized, possessing a thermal expansion coefficient of 3.25 × 10^−6^ 1/K, closely aligning with the thermal expansion coefficient of silicon wafers at 3.3 × 10^−6^ 1/K. This minimal disparity significantly diminishes thermal mismatch stress, thereby enhancing the thermal stability of the accelerometer’s output [17]. Heavily doped P-type 6-inch (100) silicon wafers are employed. The thicknesses of the silicon and glass wafers are 480 ± 20 μm and 500 ± 20 μm, respectively.

**Figure 5:**
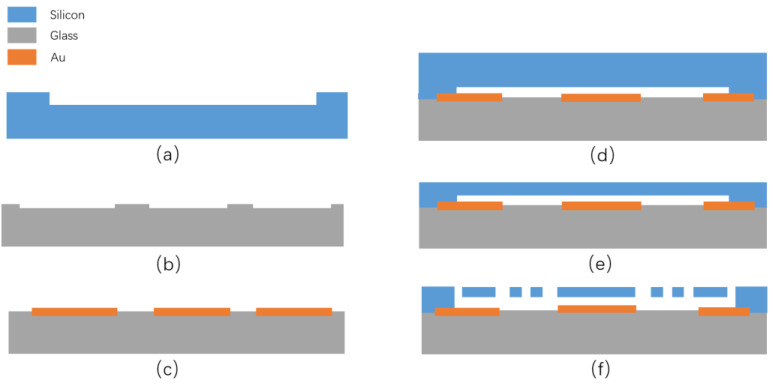
SOG process flow chart: (**a**) etching of the back cavity of the silicon wafer; (**b**) etching of the groove of the glass wafer; (**c**) fabrication of metal electrode; (**d**) the anodic bonding step; (**e**) thinning of the silicon wafer; (**f**) Etching of comb finger and spring structures.

The SOG procedure starts with a deep reactive ion etching (DRIE) to form a back cavity on the silicon wafer, as depicted in Figure 5a. This step necessitates a slow etching technique alongside a meticulous regulation of the back cavity depth as this cavity significantly influences the damping characteristics of the accelerometer. In this work, the cavity depth is controlled to 7 ± 0.3 μm. This is achieved with a DRIE system (STS-HRM) under the alternating SF_6_/C_4_F_8_ ech/passivation gases, the RF power of 800 W, and the chamber pressure at 10 mtorr; the etching rate is about 700 nm/min. 

Subsequently, a 20:1 Buffered Oxide Etch (BOE) solution is employed to form grooves on the glass substrate for accommodating the metal electrodes [18,19], as illustrated in Figure 5b. During this process, a 3 μm thick AZ5214 photoresist serves as the etch mask, and the photoresist must be appropriately hardened (10 min at 120 °C) to mitigate lateral etching by the BOE solution. In this step, the glass etching rate by this BOE solution is 29~31 nm/min and the etching depth of the glass grooves is 195 ± 10 nm.

The third step involves fabricating the metal electrodes, as shown in Figure 5c. The predominant deposition methods utilized for electrode fabrication include evaporation and sputtering. In the evaporation technique, the metal is sublimated from the source material onto the substrate in a manner akin to radiation, offering commendable directivity. Conversely, during the sputtering process, the metal particles possess substantial energy and are dispersed throughout the vacuum chamber, resulting in a lack of directivity. This directional aspect is crucial as it affects the deposition characteristics of the metal film on the sidewalls of the photoresist during the metallization process. Notably, sputtering can lead to the entire surface of the photoresist being covered, complicating the stripping procedure. Consequently, the preferred method in this context is the evaporation coating technique. In this work, a ULVAC evaporation system (model: Ei-5z) is employed, and 10 nm thick Cr and 225 nm thick Au are evaporated.

The fourth step is the anode bonding of the silicon and glass (refer to Figure 5d). The key considerations during this procedure include ensuring that both bonding surfaces are meticulously cleaned and free from any visible contaminants prior to the bonding process. The grooves made in the second step reduce the height of the metal electrodes, which greatly increases the success rate of anode bonding. In this step, a SUSS bonding system (model: CB6L) is employed. The key process parameters are as follows: the bonding temperature is 330 °C, the bonding pressure is 750 mbar, the applied voltage is 1000 V, and the bonding time is 15 min.

The fifth step is to thin and polish the silicon area of the bonded wafer, which defines the thickness of the device (Figure 5e). Important considerations for this procedure include: the following: As the silicon wafer approaches a thickness of 100 μm, the likelihood of breakage significantly escalates, necessitating a decrease in the thinning rate. Furthermore, during the structural design process, the silicon thickness should not be specified below 40 μm. In this step, the employed polishing system is AP-380F made by AM Technology, Shenzhen, China. The slurry (Trojan, Suzhou, China) is composed of silica particles in a sodium hydroxide solution. The rotation speed of the motor is set at 70 rpm, and the polishing time is 60 min.

**Figure 6:**
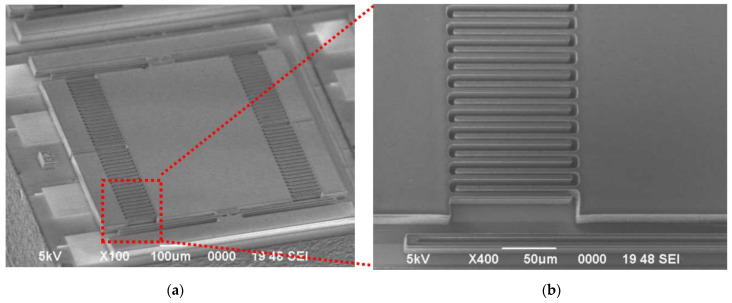
(**a**) SEM photograph of the accelerometer; (**b**) SEM photograph of comb fingers.

In the final step, a DRIE system (HSE200S from NAURA, Beijing, China) is employed to precisely etch the comb finger and torsional spring structures of the accelerometer (see Figure 5f). The etching rate is about 5 μm/min, and the etching time is 9.5 min. A slight overetching is intended to ensure the complete release of all of the microstructures. Table 3 shows the detailed configuration of the DRIE system. The suspended microstructures of the accelerometer feature fewer heat conduction pathways, resulting in a slower heat dissipation rate. The etching process necessitates close contact between the silicon wafer and the etching tray to enhance heat dissipation efficiency. Figure 6 shows the overall configuration of the accelerometer and the comb finger structure, as seen through a scanning electron microscope (SEM).

This modified SOG process reduces the thickness of the electrode between the glass wafer and the silicon wafer by etching grooves in the glass in advance to hold the metal electrode, thus greatly improving bonding success and ensuring the conductivity of the electrode.

## 3. Experimental Results

Several tests are performed to characterize the accelerometer, including frequency response test, quasi-static response test, noise measurements, and stability assessments.

### 3.1. Mechanical Test

The mechanical evaluation of the fabricated accelerometers primarily focuses on the resonant frequency along the sensitive axis. Four techniques exist for measuring this frequency [20]. The first involves utilizing an on-chip self-test unit, where the comb driver initiates the accelerometer structure through an external drive signal. By scanning the drive signal’s frequency and monitoring the sensor’s output, one can directly obtain the resonant frequency.

The second technique also employs electrostatic driving through an external signal, resembling the first approach. However, here, resonant frequency assessment relies on displacement or velocity measurements typically achieved through optical interferometry. In the third method, operators apply external acceleration directly to the accelerometer, often upon a shaker table, sweeping the acceleration frequency to extract the resonant frequency from the accelerometer’s output. The last method is to apply external acceleration to the microstructure and measure the velocity and displacement response optically.

**Figure 7:**
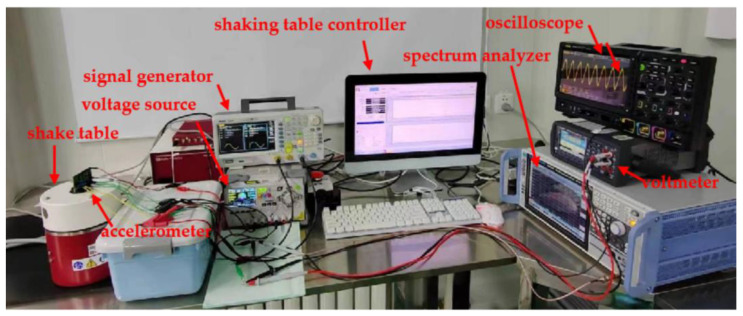
Dynamic test system.

**Figure 8:**
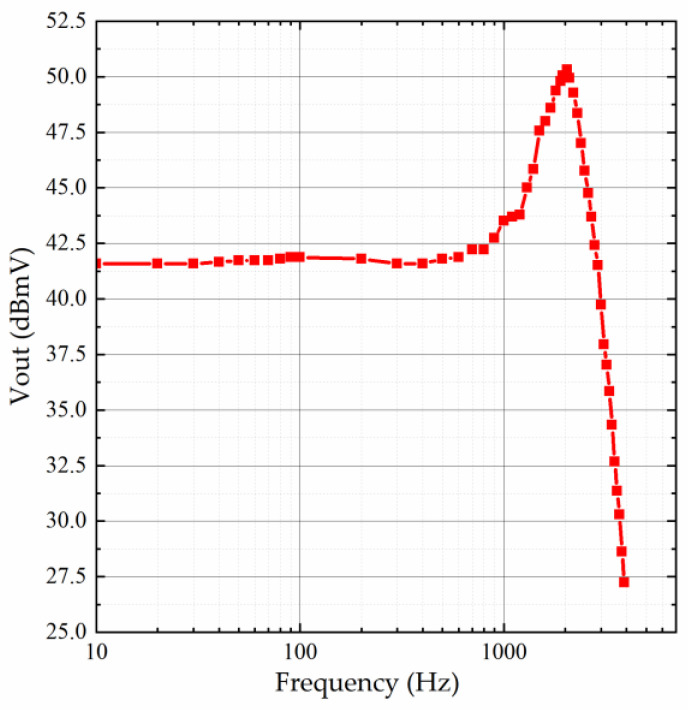
Frequency response of the accelerometer.

After careful consideration, we selected the third method for resonant frequency measurement. The test system is shown in Figure 7, where a shaker with its controller (DP901 from Data Physics, NC, USA), a spectrum analyzer (FSVA3000 from Rohde & Schwarz, Munich, Germany), a signal generator (DG4000 from RIGOL, Beijing, China), a digital oscilloscope (MSO8000 from RIGOL, Beijing, China), a digital multimeter (34465A from Keysight, Beijing, China), and a DC power supply (DP800 from RIGOL, Beijing, China) are employed. A constant amplitude for the input acceleration is applied and the accelerometer’s output is recorded when the input acceleration’s frequency is swept. The results are plotted in Figure 8. The resonant frequency of the accelerometer is 2050 Hz. The quality factor is about 2.73. This low Q outcome is resulted from the strong squeeze-film damping within the sensing microstructures [21].

### 3.2. Quasi-Static Response Test

**Figure 9:**
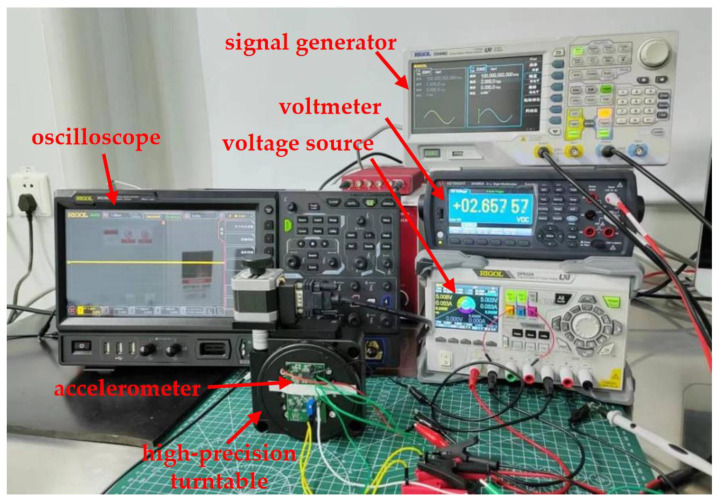
Quasi-static test system.

**Figure 10:**
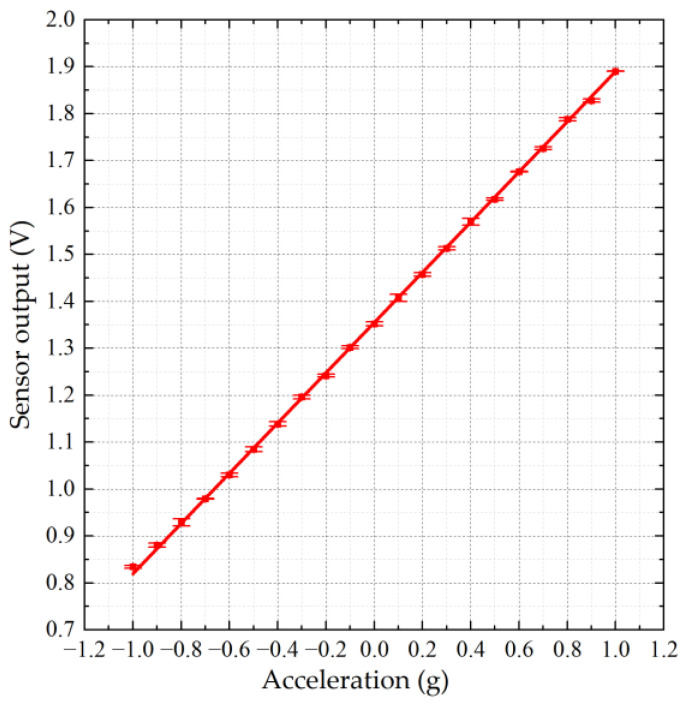
Quasi-static response of the accelerometer.

The quasi-static response test setup is shown in Figure 9, where the accelerometer is fixed on a high-precision turntable (P318 from PDV, Beijing, China). By rotating the turntable from 0° to 180°, the gravitational acceleration the accelerometer experiences along its sensing axis is changed from −1 g to +1 g. Figure 10 depicts the quasi-static response of the accelerometer. The sensitivity reaches 532 mV/g with a bias voltage of 1.5 Vpp, and the nonlinearity is less than 0.5%.

### 3.3. Noise Measurements

The noise floor of an accelerometer determines the threshold for detecting minimal acceleration. It comprises thermomechanical noise from the sensor and electrical noise from the interface circuitry [22]. The expression for noise is as follows:

(16) $$a_{\mathrm{noise}}=\sqrt{a_{m}^{2}+a_{e}^{2}}$$

where *a_n_*, *a_m_*, and *a_e_* are the overall noise, thermomechanical noise, and circuit noise respectively.

Squeeze-film damping must be considered for the designed structure with large number of lateral sensing comb fingers. The squeeze-film damping coefficient of a single pair of the comb fingers is given by [21]

(17) $$b=7.2N\mu T{\left(\frac{L_{\mathrm{finger}}}{d}\right)}^{3}$$

where *N* is the number of comb fingers, *µ* is the viscosity of the air (1.54 × 10^−6^ kg/m/s), *L_finger_* is the length of the comb fingers, *d* is the width of the gap between the comb fingers, and *T* is the thickness of the structure.

The thermomechanical noise *a_m_* can then be expressed as [23]

(18) $$a_{m}=\frac{\sqrt{4k_{B}\mathrm{tb}}}{9.8m}=9\;\mu g/\sqrt{\mathrm{Hz}}$$

where *k_B_* is the Boltzman’s constant (1.38 × 10^−23^ J/K), *t* is the absolute temperature of the working ambiance, and *m* is the proof mass of the accelerometer.

**Figure 11:**
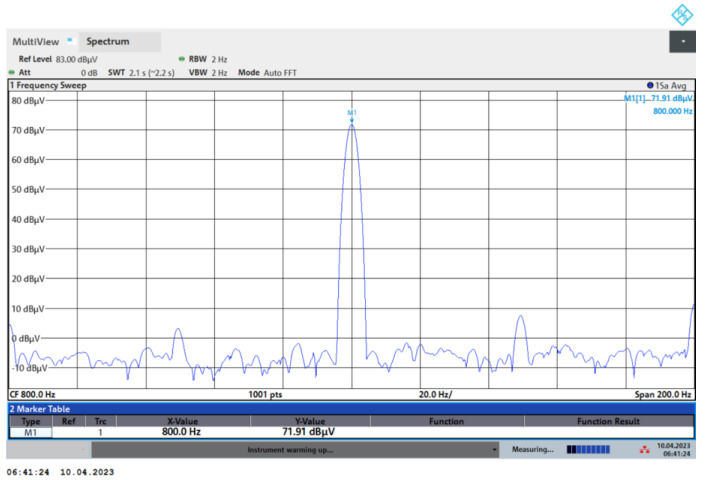
Spectrogram of the accelerometer output.

We characterized the overall noise *a_n_* using the FSVA3030 spectrum analyzer from Rohde & Schwarz, Munich, Germany. For an input acceleration of 0.5 g at 800 Hz, the power spectral density of the accelerometer output is plotted in Figure 11. It can be seen that the difference between the acceleration signal of 0.5 g and the noise floor is about 76 dB. The overall noise calculation formula of the accelerometer is as follows:

(19) $$a_{n}=\frac{0.5/\sqrt{2}}{10^{\frac{76}{20}}\mathrm{RBW}}=28\;\mu g/\sqrt{\mathrm{Hz}}$$

where *RBW* is the resolution bandwidth. *RBW* = 2 Hz.

Reducing the noise floor can be started from the squeeze-film damping of the comb finger and the noise of the interface circuit.

### 3.4. Zero-Bias Instability

**Figure 12:**
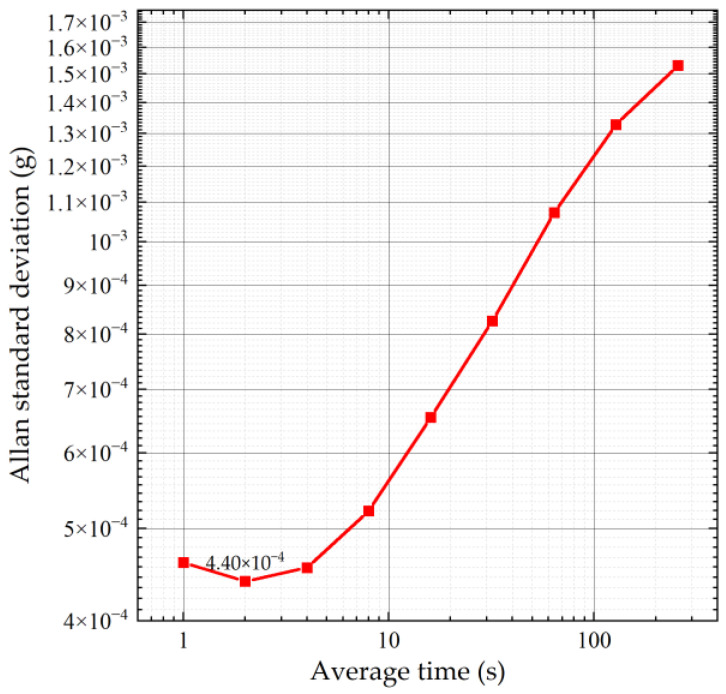
Allan standard deviation curve.

To assess the zero-bias stability of the accelerometer, we applied an input acceleration of 0 g, ensured the accelerometer remained stationary, and documented its output over a duration of approximately 6 h at ambient temperature. We employed the Allan variance analysis technique to analyze the data [24]. Figure 12 shows the Allan standard deviation curve. The figure indicates that the zero-bias instability of the accelerometer measures at 0.44 mg.

The factors contributing to this instability include material relaxation in the sensor’s microstructure, circuit gain variations, power supply fluctuations, and environmental alterations. Given the unsealed nature of the accelerometer’s package, humidity and room temperature likely serve as the primary sources of the offset drift [25]. The future development of the accelerometer will require a hermetically sealed package and a controlled testing environment to accurately identify the causes of the offset drift.

## 4. Performance Comparison

Our findings reveal two significant trends. Firstly, with *p* held constant, the capacitive sensitivity increases as the comb finger gap of the accelerometer decreases. Secondly, with *d* held constant, there is a peak in the capacitive sensitivity at a specific *p* value.

In this paper, we define the expression of capacitance sensitivity per unit area (*CSPA*) as follows:

(20) $$\mathrm{CSPA}=\frac{S_{c}d^{2}}{A_{s}}$$

where *S_c_* is the capacitive sensitivity, *A_s_* is the area of the chip, and *d* is the width of the gap between the comb fingers.

Table 4 shows a comparison of this work to the designed accelerometer reported recently in the literature. Both of the dimension and performance parameters are compared. The devices presented in this work have a compact footprint, high sensitivity, and high *CSPA*. By comparing *CSPA*, the effectiveness of this optimization method can be proved.

## 5. Conclusions

In this paper, we propose an optimal comb finger gap design for capacitive accelerometers with unequal finger gaps. By optimizing the ratio of the gaps of a rotor finger to its two adjacent stator fingers, the sensitivity of the accelerometer is maximized for the same comb finger area. Consequently, the accelerometer developed in this work exhibits superior sensitivity and a more compact design. Such advancements may benefit applications that demand high sensitivity and small size, including microrobots and wearable medical devices. In addition, we present an improved SOG process. Utilizing the refined process, we successfully manufactured the SOG capacitive accelerometer. Notably, the improved SOG process significantly elevates yield rates and has potential applications in other SOG sensor manufacturing processes. In the stability testing experiment, we observed fluctuations in the acceleration output. Thus, stability emerges as a critical factor in the future effort of improving this accelerometer design.

## Figures and Tables

**Table 1:** The capacitive sensitivity at different gap ratios.

**Gap ratio *D/d***	2	2.5	5	10	12	15
***S_c_* (F/g)**	5.78 × 10^−15^	5.97 × 10^−15^	5.24 × 10^−15^	5.18 × 10^−15^	5.16 × 10^−15^	5.13 × 10^−15^

**Table 2:** Dimensions of the accelerometer design.

Parameter	Symbol	Values
Overall sensor size	*As*	1000 μm × 950 μm
Thickness of the structure	*T*	45 μm
Comb finger gap/anti-finger gap	*d/D*	3 μm/7.5 μm
Sensing fingers	*L_finger_* × *W_finger_*	120 μm × 4 μm
Number of comb finger	*N*	42
Proof mass size	*L_mass_* × *W_mass_*	750 μm × 560 μm
Spring dimension	*L_spring_* × *W_spring_*	410 μm × 4 μm

**Table 3:** The etching parameter settings for the DRIE system.

Procedure	Cavity Pressure	ICP Power	LF Power	C_4_F_8_	SF_6_	Time
	mtorr	W	W	sccm	sccm	s
Passivation	30	1800	0	150	0	2
Etching-1	60	2200	300	0	200	1
Etching-2	60	2200	60	0	200	3

**Table 4:** Performance comparison.

References	Sensor Area (mm^2^)	Comb Finger Gap (μm)	Capacitive Sensitivity (fF/g)	Resonant Frequency (Hz)	Electrical Sensitivity (mV/g)	Noise Floor (μg/√Hz)	Zero-Bias Instability (g)	*CSPA*
This work	0.95	3	5.4	2050	532	28	4.4 × 10^−4^	51.15
Li et al. [13]	2.48	1	80	4270	35.93	NA	NA	32.25
Wang et al. [26]	77.44	4	178	400	3..47	40	8.24 × 10^−6^	36.77

## Data Availability

The datasets presented in this article are not readily available because the data are part of an on-going study.
